# Cytocidal Effects of Interstitial Photodynamic Therapy Using Talaporfin Sodium and a Semiconductor Laser in a Rat Intracerebral Glioma Model

**DOI:** 10.3390/biomedicines12092141

**Published:** 2024-09-20

**Authors:** Yuki Saito, Shinjiro Fukami, Kenta Nagai, Emiyu Ogawa, Masahiko Kuroda, Michihiro Kohno, Jiro Akimoto

**Affiliations:** 1 Department of Neurosurgery, Tokyo Medical University, Tokyo 160-0023, Japan; yuki2712@tokyo-med.ac.jp (Y.S.); k-nagai@tokyo-med.ac.jp (K.N.); mkouno@tokyo-med.ac.jp (M.K.); jiroaki@gmail.com (J.A.); 2 Department of Electronics and Electrical Engineering, Faculty of Science and Technology, Keio University, Yokohama 223-8522, Japan; emiyu@elec.keio.ac.jp; 3 Department of Molecular Pathology, Tokyo Medical University, Tokyo 160-8402, Japan; kuroda@tokyo-med.ac.jp; 4 Department of Neurosurgery, Kohsei Chuo General Hospital, Tokyo 153-8581, Japan

**Keywords:** interstitial photodynamic therapy, talaporfin sodium, malignant glioma, optical fiber, apoptosis

## Abstract

This preclinical study was conducted to investigate the efficacy of interstitial PDT (i-PDT) for malignant gliomas arising deep within the brain, which are difficult to remove. C6 glioma cells were implanted into the basal ganglia of rats, and 3 weeks later, the second-generation photosensitizer talaporfin sodium (TPS) was administered intraperitoneally. Ninety minutes after administration, a prototype fine plastic optical fiber was punctured into the tumor tissue, and semiconductor laser light was irradiated into the tumor from a 2-mm cylindrical light-emitting source under various conditions. The brain was removed 24 h after the i-PDT and analyzed pathologically. The optical fiber was able to puncture the tumor center in all cases, enabling i-PDT to be performed. Histological analysis showed that tumor necrosis was induced in areas close to the light source, correlating with the irradiation energy dose, whereas apoptosis was induced at some distance from the light source. Irradiation using high energy levels resulted in tissue swelling from strong tumor necrosis, and irradiation at 75 J/cm^2^ was most suitable for inducing apoptosis. An experimental system of i-PDT using TPS was established using malignant glioma cells transplanted into the rat brain. Tumor cell death, which correlated with the light propagation, was induced in tumor tissue.

## 1. Introduction

Glioblastoma, which is the most common type of malignant brain tumor, is a rare disease occurring in about 3 per 100,000 people [1,2]. The prognosis is highly unfavorable, with a median overall survival (m-OS) of 14.6 months and a 5-year survival rate of 9.8%, even with the standard treatment of maximal surgical resection, 60-Gy radiotherapy, and chemotherapy [3]. The rate of tumor removal by craniotomy is one of the most important prognostic factors, and for glioblastoma, a removal of more than 78% is considered to be the threshold for a comparatively favorable prognosis [4,5,6,7,8]. The prognosis for patients with residual tumors of 10 cm^3^ or less after surgical removal is 32% at 6 months, whereas for tumors of 15 cm^3^ or more, it is 3% at 6 months [9]; hence, the prognosis is highly unfavorable in patients in whom surgical removal is not feasible.

Photodynamic therapy (PDT) is a novel therapy being investigated for these malignant gliomas with a highly unfavorable prognosis. PDT, which has been the subject of clinical research since the 1980s, is a method of treatment in which a photosensitizer (PS), administered either orally or intravenously, is selectively taken up by tumors. By exposing the tumor to laser light of a specific wavelength, the PS taken up by the tumor cells is excited and the dissolved oxygen in the tissue is converted into highly toxic singlet oxygen, thereby producing an anti-tumor effect [10,11,12,13,14,15,16,17]. In Japan, an investigator-initiated clinical trial of PDT for malignant brain tumors using the second-generation PS, talaporfin sodium (TPS), started in 2009 and demonstrated an m-OS of 24.8 months and a median progression-free survival (m-PFS) of 12 months in patients with newly diagnosed glioblastoma, leading to its insurance approval in 2013 [17,18,19]. Subsequent reports by Nitta et al. also showed an m-OS of 27.4 months and an m-PFS of 19.6 months, surpassing previous treatment results, demonstrating the efficacy of PDT for newly diagnosed glioblastoma [20]. However, the current conventional method of PDT is to apply laser irradiation to the resection cavity wall after maximal safe resection [17,18,19,20], which makes it difficult to perform PDT in difficult-to-resect areas such as the basal ganglia and brainstem, and hence, does not contribute to improving the prognosis of these patients. Indeed, the m-OS for patients with high-grade basal ganglia gliomas is 10.0 months [21,22]; for those with diffuse intrinsic pontine gliomas, this is 9 to 16 months [22,23,24,25], with a 1-year survival rate of 34.9% [25]; and for glioblastomas, which are very severe, the m-OS is 6.7 months [26]. Increasing the removal rate of tumors in this region to improve prognoses as much as possible is extremely difficult in terms of preserving neurological function, with reports of adverse events resulting from surgery in as many as 34% of patients [27,28]. In other words, in cases of patients with malignant gliomas in the basal ganglia or brainstem, at present, surgery is performed only for a histological diagnosis and no treatment has yet been developed that can improve the prognosis.

Many researchers have investigated treatments using PDT to improve the prognosis of malignant gliomas arising in brain regions that are difficult to treat. The most promising of these is intra-tissue PDT (interstitial PDT: i-PDT), in which a thin fiber is punctured into the tumor and laser light is emitted from inside the tumor tissue. Although the optical delivery and irradiation protocol of PDT using fine fibers requires full investigation, the efficacy of i-PDT using a hematoporphyrin ester for malignant gliomas arising in the basal ganglia has already been reported by Kaneko in Japan [29]. Furthermore, in Germany, a prospective clinical study was reported on the efficacy and safety of i-PDT for recurrent malignant gliomas, in which 5-aminolevulinic acid was administered and an optical fiber called a laser diffuser was punctured into the tumor at several points [30,31,32]. Each of these reports demonstrates the potential of this new treatment for malignant gliomas arising in difficult-to-resect regions.

We previously investigated the potential of i-PDT with the photosensitizer TPS for malignant gliomas using a C6 glioma subcutaneous tumor model in nude mice [33]. We were able to successfully estimate the optimal timing of i-PDT from the administration of TPS, develop a fiber device for the i-PDT, and determine the optimal conditions for i-PDT to cause tumor tissue injury [33]. In the present study, we report the establishment of a model in which C6 glioma cells were transplanted into the brains of immunocompetent rats, to create a model that more closely mimics glioblastoma patients than the nude mouse subcutaneous tumor model, and to investigate whether i-PDT is as effective in this model as in the subcutaneous transplant model.

## 2. Material and Methods

### 2.1. Materials

#### 2.1.1. Cell Culture

Culture medium was prepared by adding 50 mL of heat-inactivated 10% fetal bovine serum (Gibco Thermo-Fisher Scientific, Waltham, MA, USA) and 50 U/mL penicillin–streptomycin solution (Sigma-Aldrich Co., LLC., St. Louis, MO, USA) to 500 mL of Dulbecco’s Modified Eagle Mediu (Gibco Thermo-Fisher Scientific). The C6 rat glioma cell line (Riken Cell Bank, Tsukuba, Ibaraki, Japan) was cultured in this culture medium in a 10-cm diameter dish in a CO_2_ incubator (5% CO_2_ concentration, 37 °C). The cells became confluent in about 3 days, and passages were repeated as necessary.

#### 2.1.2. Rat Basal Ganglia C6 Transplantation Model

**Figure 1:**
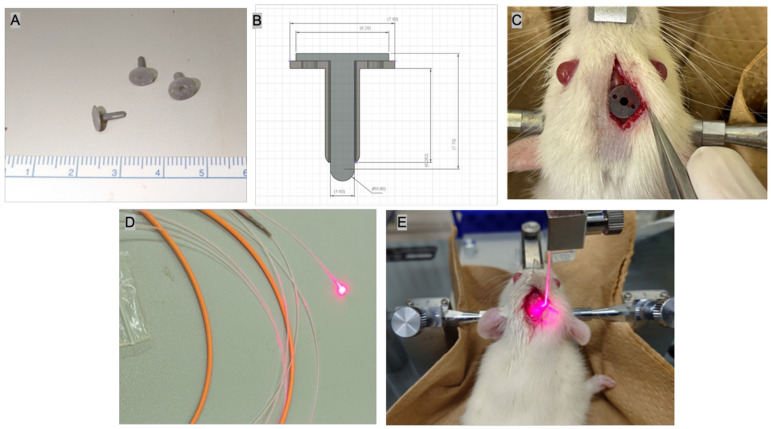
Devices and method for performing i-PDT. (**A**) Jig for accurately guiding the optical fibers, created by the Authors, using a 3D printer. (**B**) As for the size of the jig, the total length is 7.7 mm, the diameter of the fixed blade is 7 mm, and the diameter of the optical fiber passage hole is 1.6 mm. (**C**) Jig on the surface of rat brain. (**D**) A thin plastic optical fiber that emits cylindrical light with a long diameter of 2-mm, which was prepared by the Authors. (**E**) Practical application of i-PDT to rat brain tumors.

C6 glioma cells were cultured under the conditions described above and those that reached confluency were detached from the culture dish with 1 mL of trypsin (Sigma-Aldrich Co. LLC.) and collected. The resulting cell suspension was centrifuged at 27 °C, 1000 rpm, for 3 min. After removal of the supernatant, the volume was adjusted to 2 × 10^4^/µL with phosphate-buffered saline for transplantation into rats. Eight-week-old male Wistar rats (Japan SLC Inc., Hamamatsu, Shizuoka, Japan) weighing 200 g to 250 g were sedated under intraperitoneal anesthesia with a triad of anesthetics. Then, the head was fixed to a stereotactic instrument for rats (SR-5R-HT: Narishige Scientific Instruments Lab., Tokyo, Japan), a dental drill was used to puncture a point 3 mm right lateral and 2 mm anterior to Bregma, and a tumor cell suspension was injected into the striatum at a depth of 5 mm from the brain surface at this point. Ten microliters of cell suspension was slowly injected using a 27-gauge—gauge Hamilton micro-syringe, the cells were transplanted, and the mice were housed under normal conditions for 3 weeks. For the operation, a jig with a 27-gauge needle threading was fabricated to allow implantation at a certain depth from the brain surface. A special jig (created using a 3D printer) that can be implanted intracranially was used to reduce brain contusions that could occur during subsequent manipulations, while also making it easy to identify the tip of the emission site of the optical fiber on Hematoxylin and Eosin (HE) specimens (Figure 1A–C).

#### 2.1.3. Talaporfin Sodium (TPS)

TPS, with a molecular weight of 799.69, is a second-generation water-soluble PS consisting of an aspartic acid amide bonded to a chlorin backbone derived from plant chlorophyll. It is characterized by faster elimination and a shorter period of post-administration shading than the first-generation PS. Excitation wavelengths are 405 nm and 664 nm, and the latter wavelength is used for PDT as this wavelength is not affected by absorption by red blood cells and water. In Japan, TPS was first covered by insurance for early-stage lung cancer in 2004, followed by malignant glioma in 2013, and recurrent esophageal cancer after radio-chemotherapy in 2015; thereby, it became widely used in Japan as a PS for PDT.

#### 2.1.4. Laser Irradiation Fiber Probe

The fine-diameter optical fiber used for cylindrical irradiation is an original polymethyl methacrylate optic fiber (diameter: 0.8 mm; emission band: 2.0 mm; emission area: 0.0553 cm^2^; transmittance: 65%; Nissei Electric Co., Machida, Tokyo, Japan) (Figure 1D). The laser light source Rouge-LD (664 nm, 500 mW, class 4 continuous-wave laser diode, Cyber Laser Inc., Tokyo, Japan) was used. To ensure stable and accurate implantation of a thin optical fiber at the tumor center, an original experimental jig was developed using a 14 G intravenous needle (Becton Dickinson and Company, Inc., Franklin Lakes, NJ, USA) with a 3D-printed fixing blade attached with instant adhesive (Figure 1A–C). A stopper was provided behind the optical fiber to ensure that the light-emitting surface of the thin optical fiber was exposed at the tip of the outer cylinder so that the fiber could always be irradiated accurately to the tumor center.

### 2.2. Methods

#### 2.2.1. Implementation of i-PDT

Tumors grown in the right basal ganglia of disease-model rats (N = 5) were irradiated. Ten milligrams per kilogram dose of TPS was administered intraperitoneally to rats. After 90 min, an optical fiber was inserted 10 mm through the guidance hole of the dedicated jig, and red laser light at a wavelength of 664 nm was irradiated into the tumor tissue under various conditions (Figure 1E and Table 1). Groups A to C were the i-PDT groups (A: 150 J/cm^2^; B: 75 J/cm^2^; C: 50 J/cm^2^), group D was treated with intraperitoneal TPS but no laser irradiation, and group E received neither TPS nor laser irradiation. The special jig and rat scalp were firmly fixed with tissue instant adhesive and 4-0 nylon thread to prevent them from falling off (Figure 1E). During i-PDT, induction anesthesia was performed using intraperitoneal pentobarbital and inhalation anesthesia with isoflurane (Pfizer Inc., New York, NY, USA).

#### 2.2.2. Post-Irradiation Tissue Sampling

Twenty-four hours after i-PDT, the treated rats were deeply anesthetized with a triad of anesthetics and sacrificed by cervical dislocation. The cranium was opened, and the brain was removed in a single block and then fixed in 10% formalin solution. After sufficient fixation, the fiber insertion corridor was cut out in the coronal plane so that it could be observed, and the tumor surface was exposed and paraffin-embedded.

#### 2.2.3. Histopathological Analyses

Morphological assessments were performed by HE staining. To investigate the tumor-killing effect of i-PDT using TPS, the TUNEL staining method was used (in situ apoptosis detection kit MK500, Takara Bio, Tokyo, Japan). The label was a rabbit-derived anti-fluorescein isothiocyanate horseradish peroxidase conjugate with 10× dilution of terminal nucleotidyl transferase, and the chromogenic substrate was a 50× dilution of 3,3′-diaminobenzidine and the ApopMark™ Apoptosis Detection Kit (Exalpha Biologicals Inc., Maynard, MA, USA). Phospho-tungstic acid hematoxylin (PTAH) staining and immunostaining with an anti-CD31 antibody were used to assess thrombi in tumor vessels. For further immunological evaluation, immunohistochemical analyses were performed using monoclonal antibodies for CD4, CD8, CD20, GFAP, Iba-1, TP53, and CD68 (Abcam plc., Cambridge, UK). Analysis of each virtual slide was performed using NDP.viewer2 U12388-01 (Hamamatsu Photonics K.K., Hamamatsu, Shizuoka, Japan). For specimens stained with the ApopMark™ DNA Fragmentation Detection Kit, 10 locations around the tip of fiber used for laser irradiation of the tumor were arbitrarily selected in the 200× field-of-view of sections from each condition, and the number of cells positive for staining was counted using Patholoscope software (Mitani Co., Tokyo, Japan) to determine whether there are significant differences between groups.

#### 2.2.4. Statistical Processing

All data were analyzed using IBM SPSS Statistics 29.0.2 software (Statistical Package for the Social Sciences; IBM Japan, Ltd., Tokyo, Japan). Statistical analysis was performed by one-way analysis of variance (ANOVA), and a *p*-value less than 0.05 was considered to indicate a statistically significant difference between groups.

## 3. Results

### 3.1. C6 Transplantation Rat Basal Ganglia Glioma Model

In all rats, a tumor with a mass of 3 to 5 mm was formed in the right basal ganglia, with tumor cell infiltration at the border with the normal brain. Necrosis and hemorrhagic changes were observed in the center of the tumor, and the cells comprising the tumor were markedly atypical, with abundant mitotic figures and neovascularization with the proliferation of endothelial cells. Large tumors were also formed with edema in the surrounding brain and a strong deviation of the median structure, closely mimicking the glioblastoma pathology observed in clinical practice.

### 3.2. Tumor Pathology 24 h after i-PDT

**Figure 2:**
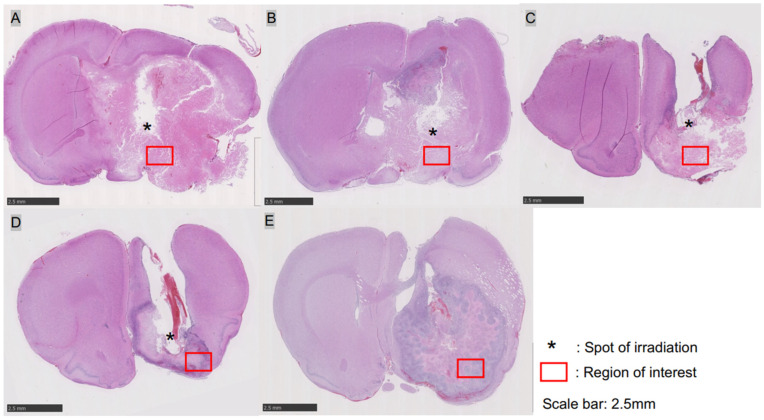
HE-stained macroscopic view of C6 glioma tissue after undergoing i-PDT of various conditions: (**A**) 150 J/cm^2^; (**B**) 75 J/cm^2^; (**C**) 50 J/cm^2^; (**D**,**E**) control. In group A, tumor tissue swelling occurred along with extensive destruction of tissue owing to i-PDT, resulting in the deviation of the midline structures. In groups B and C, extensive destruction of tumor tissue occurred, except in the spots of light irradiation (asterisk). Group D of the control showed necrosis in the center of the tumor but with viable tumor tissue around it. Group E showed massive tumor tissue with no necrosis or apoptosis. The red boxed area in each figure is the region of interest where detailed pathological analysis was subsequently performed. The scale bar for all figures indicates a length of 2.5 mm.

**Figure 3:**
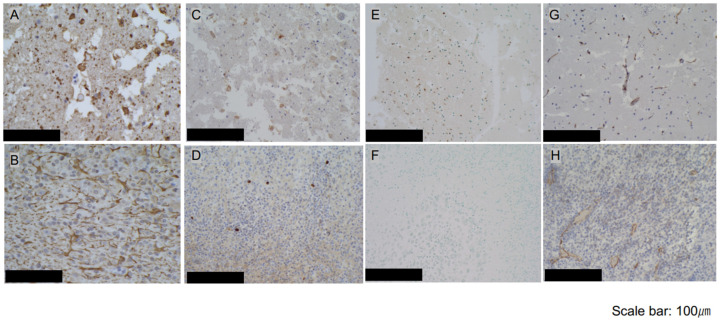
Microscopic view of C6 glioma tissue slightly distant from the light source (ROI in Figure 2) in the i-PDT (75 J/cm^2^) and control groups. (**A**,**B**) Immunostaining of the tumor tissue with an anti-GFAP antibody (100×). i-PDT resulted in shortening of tumor cell processes and condensation of their cytoplasm. (**C**,**D**) Immunostaining with an anti-p53 antibody (100×). Higher p53-positive cell rates were observed in the i-PDT-treated groups than in the control groups. (**E**,**F**) Assessment of apoptosis by TUNEL staining (100×). More TUNEL-positive apoptotic cells were observed in the i-PDT-treated groups than in the control groups. (**G**,**H**) Immunostaining with an anti-CD31 antibody (100×). Narrowing of the vascular bed of CD31-positive tumor vessels was observed in the i-PDT-treated groups. The scale bar for all figures indicates a length of 100 μm.

HE staining demonstrated tumor-destructive changes in each of the i-PDT irradiation conditions, although there were some differences. In group A (150 J/cm^2^)—in which tumors were irradiated with the highest energy density—tumor tissues showed extensive necrotic changes, combined with edematous changes in the surrounding brain, demonstrating a deviation of the median structure. Extensive necrotic changes in tumor cells were also induced in groups B (75 J/cm^2^) and C (50 J/cm^2^), but residual tumor tissues were observed, with no edematous changes that could cause a deviation of the midline structure. The control groups D and E also showed necrotic changes in the tumor tissue, but only in some parts of the tissue, and did not show extensive tumor tissue necrosis as observed in the i-PDT group (A–C) (Figure 2). In the assessment of intra-tumor vessels, fibrin thrombus formation as identified by PTAH staining was not observed in the i-PDT-treated groups (A–C) but CD31 immunostaining gave the impression of a narrow vascular bed in the i-PDT-treated groups that was not observed in the control group (D, E) (Figure 3G,H).

Various immunostaining evaluations were performed, and staining with an anti-GFAP antibody revealed scattered tumor cells in the i-PDT group outside of the necrotic area around the light source, which had a spherical morphology with shortened tumor cell processes and a condensed cytoplasm (Figure 3A,B). Staining with a TP53 antibody demonstrated a high rate of positive cells in the same area (Figure 3C,D). Evaluation of apoptosis by TUNEL staining showed numerous positive cells outside the necrotic area around the light source in the i-PDT-treated groups but almost no positive cells in the control groups (Figure 3E,F). No significant differences were found between the i-PDT and control groups in terms of the positivity rate of immunocompetent cells, such as those positive for CD4, CD8, Iba-1, and CD68.

### 3.3. Quantitative Assessment of Apoptosis with ApopMark™

**Figure 4:**
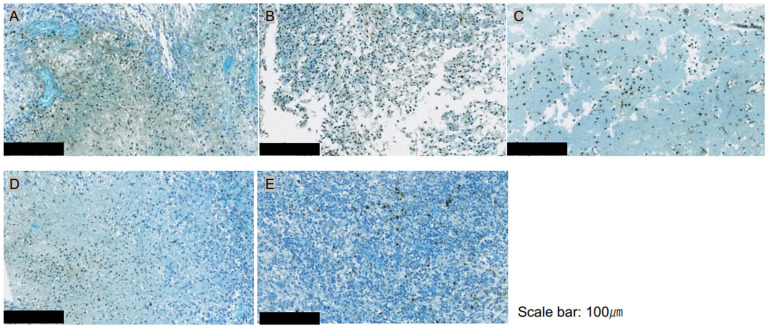
Apoptosis assessment of i-PDT-treated (**A**–**C**) and control (**D**,**E**) groups in ROIs of Figure 2 by ApopMark™ (100×). More ApopMark™-positive apoptotic cells are seen in the i-PDT groups than in the control groups, with the highest positive cell rate in the 75 J/cm^2^ treatment group (**B**). The scale bar for all figures indicates a length of 100 μm.

**Figure 5:**
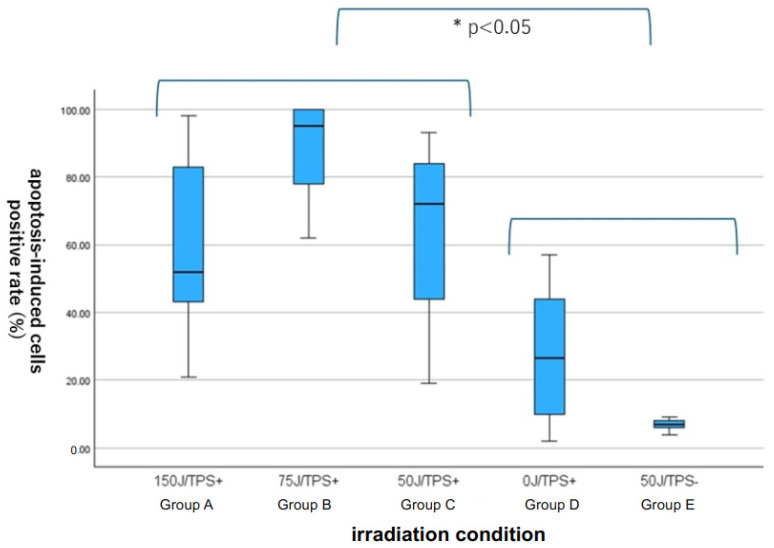
Comparison of the number of ApopMark™-positive cells in the i-PDT-treated groups (**A**–**C**) and the control groups (**D**,**E**). There were statistically significantly more apoptotic cells in the i-PDT-treated groups than in the control groups but there were no statistically significant differences among the 3 i-PDT-treated groups.

Specimens stained with ApopMark™ from the i-PDT-treated groups (A: 150 J/cm^2^: B: 75 J/cm^2^, and C: 50 J/cm^2^) and the control groups were captured as virtual slides, and 10 regions of interest were randomly selected from around the optical diffuser of the laser-irradiated fiber. The number of apoptosis-induced cells in the 200× field-of-view was measured using Patholoscope software. The results showed that the i-PDT-treated groups had significantly more apoptosis-induced cells than the control groups, with the 75 J/cm^2^ irradiation group having the most apoptosis-induced cells among i-PDT-treated groups (A–C), although no statistically significant differences were observed (Figure 4 and Figure 5).

## 4. Discussion

PDT, a treatment for malignant brain tumors, is considered to be an additional treatment that targets residual infiltrating cells after maximal safe resection by craniotomy, and there have been many reports on its clinical efficacy and safety [17,18,19,20,34,35,36]. In other words, at present, PDT is only indicated for patients in whom near-total removal of the tumor is judged to be possible before surgery. However, a review of the location of 2049 glioblastomas reported in the Japanese Brain Tumor Registry (2005–2008) showed that 61 glioblastomas were located in the basal ganglia, 85 in the thalamus and hypothalamus, and 38 in the brainstem [37]. This means that 184 (8.98%) glioblastomas occurred in these difficult-to-resect sites, and these cases were contra-indicated for PDT in the preoperative diagnosis. However, we have been investigating whether these patients with glioblastoma that occur in these site—which constitute less than 10% overall—could also benefit from PDT. We hence investigated the possibility of i-PDT as an alternative to conventional PDT, which irradiates the surface of the tumor resection cavity.

We previously demonstrated that PDT using TPS has tumor-cell-killing effects on several malignant glioma cell lines in an in vitro study, in a TPS concentration- and time-dependent manner after PDT [38,39,40,41,42]. It has also been reported that the cell-killing effect is essentially the result of both tumor cell necrosis and apoptosis caused by PDT-induced singlet oxygen toxicity, with lysosome and mitochondrial crosstalk in the tumor cytoplasm playing an important role [39,40,42]. Furthermore, in vivo experiments of surface-irradiated PDT in a rat brain tumor model in which C6 cells were implanted on the surface of the brain have shown that surface irradiation induces necrosis of tumor tissue on the surface of the brain close to the light-irradiated surface, and apoptosis deep within the brain, with the effect being enhanced over time [43]. In other words, it was speculated that the induction of a cell-killing effect similar to surface irradiation could be achieved for deep-brain tumors if light irradiation could be applied precisely after TPS administration.

The problem was how to deliver the light into the tumor tissue. We therefore used a light diffuser made of plastic optical fibers with a diameter of 250 µm, which had already been developed by co-authors Ogawa et al. [44]. This fiber has a 0.8-to-1-mm diameter fluorinated resin material was used as the outer structure, which is useful from the viewpoint of biocompatibility and heat resistance, through which a thin plastic optical fiber is threaded through to ensure rigidity to puncture the tumor. The light diffuser has a diffusion length of 2 mm to enable full circumferential irradiation, and is constructed to create an air layer between the fibers and the outer tube to reduce heat generation owing to prolonged irradiation [44]. In our previous study, we established an experimental system of i-PDT in a nude mouse C6 subcutaneous implant glioma model using this optical fiber and confirmed that accurate intra-tumor puncture and irradiation were possible and that tumor tissue destruction was achieved, correlating with the irradiation light energy intensity [33]. Therefore, it was concluded that it would be possible to perform PDT experiments with full circumferential irradiation of the tumor center using this optical fiber in the present intracerebral transplantation model.

We found that the fiber could be punctured precisely into the center of the tumor in the basal ganglia C6 transplantation model, and i-PDT could be performed under various light irradiation conditions. Furthermore, in the area close to the i-PDT light source—which provides full circumferential irradiation—extensive tissue necrosis was induced, whereas at some distance, apoptosis was induced, confirmed by the TUNEL staining and ApopMark™. In the aforementioned experiment on a brain surface glioma model using C6 glioma cells, the results of the laser irradiation of the tumor surface using TPS showed that of the C6 glioma tissue extending 2 mm into the brain parenchyma, extensive necrosis occurred from the surface to 1.5 mm, with 0.5 mm at the deep end remaining [43]. The apical tumor tissue had undergone apoptosis, as confirmed by M30-CytoDeath immunostaining [43]. In the present i-PDT experiments, necrosis was observed in the area close to the light source, and apoptosis was observed at some distance from the light source, suggesting there is a gradient in the effect of PDT owing to the light delivery into the brain tissue in i-PDT using TPS. In future experimental systems of i-PDT, it will be necessary to verify the consistency between light delivery and the actual degree of tissue injury.

The i-PDT group was divided into three energy density groups, i.e., 150 J/cm^2^ (group A), 75 J/cm^2^ (group B), and 50 J/cm^2^ (group C), and the pattern of tumor tissue injury was compared. In group A, extensive necrosis of the tumor tissue was observed, some cells undergoing apoptosis were found, and the midline structure was deviated owing to swelling of the tissue caused by the severe necrosis. Group B also showed necrosis around the light source, and the density of cells undergoing apoptosis in the surrounding area was the highest. Group C also showed necrosis and apoptosis but the frequency of apoptosis was not as high as in group B. To develop i-PDT for deep-seated malignant gliomas for clinical use in the future, it is necessary to consider the safety of i-PDT for the surrounding brain structures, to establish a protocol for i-PDT that does not cause severe necrosis induction owing to tumor swelling, and substantial tissue cell injury by apoptosis. In our previous experiments, in a C6 subcutaneous transplantation glioma model in nude mice, we found that when the same energy density was applied, irradiation at a low power density over a long time was more effective than a high power density over a short time in inducing apoptosis [33], and a similar tendency is thought to exist in the intracerebral transplantation model.

In our previous experiments on i-PDT, tumor tissue damage was also observed as indicated by the gradient of light propagation but we also noted that the effect of PDT on tumor vessels contributes to the tissue damage effect [33]. In other words, our results showed that thrombus formation in tumor vessels by PDT could induce secondary ischemic tissue damage [45,46]. However, in the present study using an intracerebral transplantation glioma model, no noticeable thrombus formation was observed in the vessels in the tumor tissue according to the differences in the condition settings of PDT, such as its power density and energy density. However, a study using vascular endothelial staining with CD31 antibodies showed a trend toward a narrowing of the vascular bed of intra-tumor vessels in the i-PDT-treated group. The effect of PDT on tumor vessels has generally been described as a vascular shutdown effect but there have been scattered reports of vasoconstriction and vasospasm being induced, which do not lead to shutdown, depending on the irradiation conditions [46]. Our i-PDT conditions were highly likely effective in inducing necrosis and apoptosis of tumor tissue but appeared to have minor effect on the tumor vessels. Few reports to date have discussed the effects of i-PDT on blood vessels, and this may be an area of focus for future research [45,46].

In recent years, damage-associated molecular patterns that develop in tumor cells as a result of PDT-induced tissue damage have been shown to stimulate host anti-tumor immunocompetence [47,48]. In our present experimental system, we repeatedly performed analyses by sacrificing the rats at 24 h after i-PDT but we were unable to show a clear trend in the activation of all immune systems, including T cells, B cells, and microglia. This suggests that it may be crucial to evaluate the activation of tumor immunity by i-PDT at a later timepoint than 24 h after its implementation.

## 5. Conclusions

In our experimental study of i-PDT using TPS for a C6 brain transplantation rat glioma model, our prototype thin plastic optical fiber was able to accurately guide the light source to the tumor center and deliver cylindrical laser irradiation with a diffusion length of 2 mm. Although the pattern of tissue injury varied according to the conditions under which i-PDT was performed, tumor tissue necrosis was induced in the area close to the light source, whereas apoptosis was induced at a slightly more distant site, suggesting that the light propagation gradient was consistent with tissue injury. Other effects of PDT, such as vascular shutdown effects and secondary activation of host anti-tumor immunity, may require different i-PDT protocols and future studies analyzing changes over long periods of time after i-PDT using TPS.

## Figures and Tables

**Table 1:** Conditions for performing i-PDT in each group.

Group	N	Energy Density (J/cm^2^)	Power Density (mW/cm^2^)	Input Power (mW)	Time (Second)	TPS
A	5	150	150	8.3	1025	(+)
B	5	75	73	4	1025	(+)
C	5	50	150	8.3	334	(+)
D	5	0	0	0	0	(+)
E	5	50	150	8.3	334	(−)

## Data Availability

The datasets used and/or analyzed during the current study available from the corresponding author on reasonable request.
